# The effect of mandala activity on postoperative pain, anxiety, and analgesia use in gynecologic oncology patients: a randomized controlled trial

**DOI:** 10.1007/s10552-026-02125-4

**Published:** 2026-02-02

**Authors:** Saliha Akyol, Ayça Demir Yıldırım

**Affiliations:** Department of Midwifery, Faculty of Health Sciences, Uskudar University, Istanbul, Turkey

**Keywords:** Pain, Anxiety, Midwife, Gynecologic Oncology, Mandala

## Abstract

**Purpose:**

Non-pharmacological complementary therapies in cancer treatment strengthen care. This study aims to investigate the effects of mandala activity on postoperative pain, anxiety level and analgesia use in gynaecological oncology patients.

**Materials and methods:**

This study is a randomised controlled, two-group pretest/posttest comparative study. The study included 42 patients who underwent open abdominal hysterectomy at a training and research hospital in Istanbul between 1 February and 1 August 2023. Introductory Information Form, Visual Analog Scale (VAS), State-Trait Anxiety Inventory (STAI-I), Postoperative Patient Evaluation Form and Mandala Activity Booklet were used throughout the study.

**Results:**

Preoperative State Trait Anxiety Inventory (STAI-I) score was significantly higher in the control group (63.48 ± 5.31) compared to the intervention group (56.33 ± 7.65) (*p* < .05). Postoperatively, the STAI-I score of the control group decreased to 47.43 ± 8.29, while the intervention group showed a significant decrease to 31.52 ± 6.73. In addition, the Visual Analog Scale (VAS) scores of the control group decreased from 7.17 ± 1.42 preoperatively to 0.82 ± 1.46 postoperatively, while the intervention group scores decreased from 6.68 ± 2.11 to 1.77 ± 1.79, indicating that mandala activity was significant (*p* < .05).

**Conclusion:**

In conclusion, this study showed that postoperative mandala activity effectively reduced pain and anxiety levels and influenced the use of analgesia in gynecologic oncology patients.

## Introduction

Pain and anxiety are common and important complications of the treatment process in gynecologic oncology patients. The relationship between pain and anxiety is defined as a combination of physical, psychological and social factors, which is also emphasized in the biopsychosocial model [1, 2]. Pain can directly affect the emotional state of patients and increase their anxiety levels, which can negatively affect the healing process [3]. Therefore, effective pain and anxiety management is critical for the overall recovery process of gynecologic oncology patients. Therefore, the pain and anxiety of patients with gynecologic oncology needs to be managed effectively [4]. In addition to pharmacological methods in the effective management of pain and anxiety, non-pharmacological methods are also recommended by the World Health Organization [5]. Research on non-pharmacologic methods, sampled from cancer patients, appears to be effective [3, 6]. These non-pharmacological methods include aromatherapy, acupressure, hypnosis, massage, prayer, breathing exercises, music therapy, and art therapy [3]. Art therapy appears to reduce anxiety and psychological symptoms in patients with gynecologic cancer [7, 8]. One of these art therapies is mandalas [9, 10]. Jung describes the mandala as the center that symbolizes the self and argues that this center is the womb. Mandala is applied as a therapeutic tool in psychotherapy [11]. The literature reveals that various studies have examined the relationship between mandala activities and pain in relation to pregnancy, childbirth, women’s health and diseases. [12–19]. Although existing research on the effect of mandala activities on pain is limited, there are very few studies in which the sample group consists of cancer patients [20]. A randomized controlled trial on the effect of mandala art therapy on both pain and anxiety levels in the perioperative period of gynecological oncology was not found in the literatüre [21]. Midwifery practice encompasses the continuum of women’s health needs, extending beyond pregnancy and childbirth to include reproductive health promotion and disease prevention. According to the International Confederation of Midwives (ICM), midwives have responsibilities in recognizing early signs of gynecological disorders, providing counseling, and referring women for appropriate care when necessary [22]. In this context, gynecological oncology, particularly in the areas of prevention, screening, treatment support, and early diagnosis, falls within the extended scope of midwifery care. Midwives play an essential role in providing holistic care to women diagnosed with gynecological cancers, addressing both their physical and psychological needs during treatment and recovery [23]. Mandala activity, as a type of art therapy, has been recognized as an effective non-pharmacological method for reducing anxiety and promoting emotional balance. Based on Jung’s theoretical framework, mandalas symbolize the individual’s inner self and serve as a tool for psychological relief [18]. Therefore, this study aims to explore the potential effects of mandala activity on postoperative pain, anxiety levels, and analgesia use among gynecologic oncology patients, contributing to both women’s health outcomes and the scope of midwifery practice.

The hypotheses of this study are as follows:

H1. There is a difference between the pain scale scores of the intervention group and the control group.

H2. There is a difference between the state anxiety scale scores of the intervention group and the control group.

H3. There is a difference between the number of analgesia use in the intervention group and the control group.

## Materials and methods

### Research design

This study designed as a randomized controlled, two group, pretest–posttest comparative, experimental study with 42 patients who underwent open abdominal hysterectomy surgery in the Gynecological Oncology Surgery Clinic of a training and research hospital in Istanbul, Turkey between 1 February 2023 and 1 August 2023.

### Sample

The study population consisted of patients who were hospitalized in the gynecologic oncology surgery clinic of the hospital and underwent open abdominal hysterectomy surgery. The total number of gynecologic oncology patients who underwent open abdominal hysterectomy surgery within one year was 198 cases. The sample size was calculated based on the mean anxiety scale scores of Bell at all [7]. It found to be a total of 30 people with 95% power by G Power analysis. Considering the loss of cases, it increased by 40% and a total of 42 people included in the sample. The sample randomized according to patient room numbers and divided into two groups as intervention and control groups.

For the randomization of the study, women admitted to the gynecological oncology service were numbered according to the order of admission. These numbers were entered into the randomizer.org website and divided into groups by assigning numbers to two unique groups.

Inclusion criteria required participants to have been diagnosed with a gynecologic oncology disease and to have undergone open abdominal hysterectomy surgery. Participants had to be over 18 years of age and able to communicate in Turkish. In addition, this had to be their first gynecologic oncology surgery and they had to have no physical disability related to the upper extremities. Exclusion criteria for the study were readmission due to any complication after discharge and any visual, auditory or language problems. In the study, the research conducted in accordance with randomization. Due to the nature of the study, the investigator, patients and statistical expert were not blinded.

The dependent variables of this study were pain score, anxiety scale score and analgesia use. The independent variables were marital status, family type, place of residence, educational status, employment status, occupation, income, number of living children, presence of chronic disease, diagnosis of the disease, reason for applying to the hospital, regular gynecological examination status, type of hysterectomy surgery, equipment found in the patient in the postoperative period, type of analgesia applied in the postoperative period and mandala activity.

### Data collection tools

Informed Voluntary Consent Form obtained from the entire sample group. A total of 27-question Descriptive Information Form with 16 questions about demographic characteristics in the first part and 11 questions about gynecologic characteristics in the second part was completed by the researcher. The VAS scale, a pain scale accepted in the universal literature, and the STAI-I, a state anxiety scale, were administered by the researcher. Mandala Activity Booklet consisting of 10 mandala colorings created by the researcher was applied to the intervention group. The 11-question Postoperative Period Patient Evaluation Form completed by the researcher before discharge in both groups.

### Introductory information form

It is a form developed by researchers based on the literature and consists of a total of 27 questions covering demographic characteristics such as age, educational status, employment status, and gynaecological history, including past illnesses and menstrual cycle. There are 16 questions in the demographic history and 11 questions in the gynaecological and obstetric history [24].

### Visual analog scale – VAS

This scale is used to visually assess and then quantify values that cannot be measured quantitatively. It used to measure the pain level of gynecologic oncology patients in both the intervention and control groups. In this scale, the variable to be evaluated is written on both ends of a 100 mm long line. The patient was asked to place a line, dot or cross on this beam by asking which one she was closer to. For example, the leftmost part is labeled “no pain” and the other end is labeled “most severe pain”. The patient makes a mark on the ray according to his/her current condition. Starting from the left end, i.e., where there is no pain at all, the part up to the point where the patient has pain, i.e., where the patient makes a mark, is measured. This quantity indicates the patient’s pain level. The increase in quality and quantity indicates an increase in pain level [25].

### State-trait anxiety ınventory (STAI) scal*e*

This inventory developed by Spielberger et al. in 1970 and the Turkish validity and reliability study was conducted by Öner and Le Compte in 1975. The scale measures state and trait anxiety as Likert scale with 20 separate questions. The higher the scores, the higher the level of anxiety and the lower the scores, the lower the level of anxiety. The total score of both scales ranges from a minimum of 20 to a maximum of 80. A high score indicates a high level of anxiety and a low score indicates a low level of anxiety [26]. Only the State-Trait Anxiety Inventory (STAI-I) part of the scale was used.

### Postoperative patient evaluation form

This form was prepared by the researchers and was designed to evaluate factors such as the surgery, analgesic use, the hours of pain in the postoperative period and the type of analgesic applied, sleep patterns, and satisfaction with the application [1, 7]. This form consists of 11 questions in total.

### Implementation of the research

Informed consent obtained from the randomized sample groups. Then, the Introductory Information Form, Visual Analog Scale (VAS) and State-Trait Anxiety Inventory (STAI-I) completed by the patient who had not yet undergone surgery. The sample group divided into two groups as control and intervention group according to randomization. The Postoperative Patient Assessment Form administered by the researcher to both the control and intervention groups before the patient was discharged after surgery in order to evaluate the patient’s analgesic needs in this process, and in addition, the intervention group asked questions about mandala practice.

The participants in the intervention group were given a mandala booklet prepared by the researcher. This booklet contains 10 mandala colorings selected by the researcher, each representing themes such as nature, the flower of life, energy, the atom, and the lotus flower, connecting them to concepts of the universe and birth, ranging from traditional to modern symbols [27] articipants asked to do the mandala activity by coloring the mandala drawing selected from the booklet as much as they wanted on the zeroth, first and second postoperative day during their hospital stay. However, they were asked to do at least one mandala activity every day. A 12-color dry paint set was given to the patients by the researcher for painting in the mandala activity and no color guidance was given for painting. The study followed up for three days as the zeroth, first and second postoperative days. VAS and STAI-I form filled before and after the mandala activity. In addition, the VAS form repeated every four hours until the patient was discharged.

In the control group, STAI-I and VAS forms filled out at one hour intervals during the hours when the intervention group practiced mandala activity without any intervention. In addition, the VAS form repeated every four hours in the control group until the patient was discharged. Postoperative Patient Evaluation Form was filled in both groups before discharge.

### Statistical analyses

The data analyzed using the Statistical Package for Social Sciences (SPSS) Windows 25.0 program. Descriptive statistical methods (number, percentage, min–max values, mean and standard deviation) used to evaluate the data. “Reliability Analysis” was performed to test the reliability of the scales.

The compatibility of the used data with normal distribution was tested with kurtosis and skewness values. Independent sample t test was used for the difference between two independent groups in the comparison of quantitative data in normally distributed data. The dependent sample t test was used for the difference between two dependent groups, and repeated measures analysis of variance was used for the difference between more than two groups, and the Bonferroni test used to determine the measurement from which the difference originated. The comparison of the scores according to group, time and group*time interaction was analyzed using “Two-way Analysis of Variance in Repeated Measures”. Chi-square test used for the relationship between categorical variables.

### Ethical permissions and research registration.

T.C. Istanbul Üsküdar University Non-Interventional Research Ethics Committee Approval received from the Ethics Committee of Üsküdar University on 30.12.2022 61,351,342. Written consent obtained from the participants with the Informed Voluntary Consent Form. The research registered in clinical trials with the number NCT06590389.

## Results

**Fig. 1:**
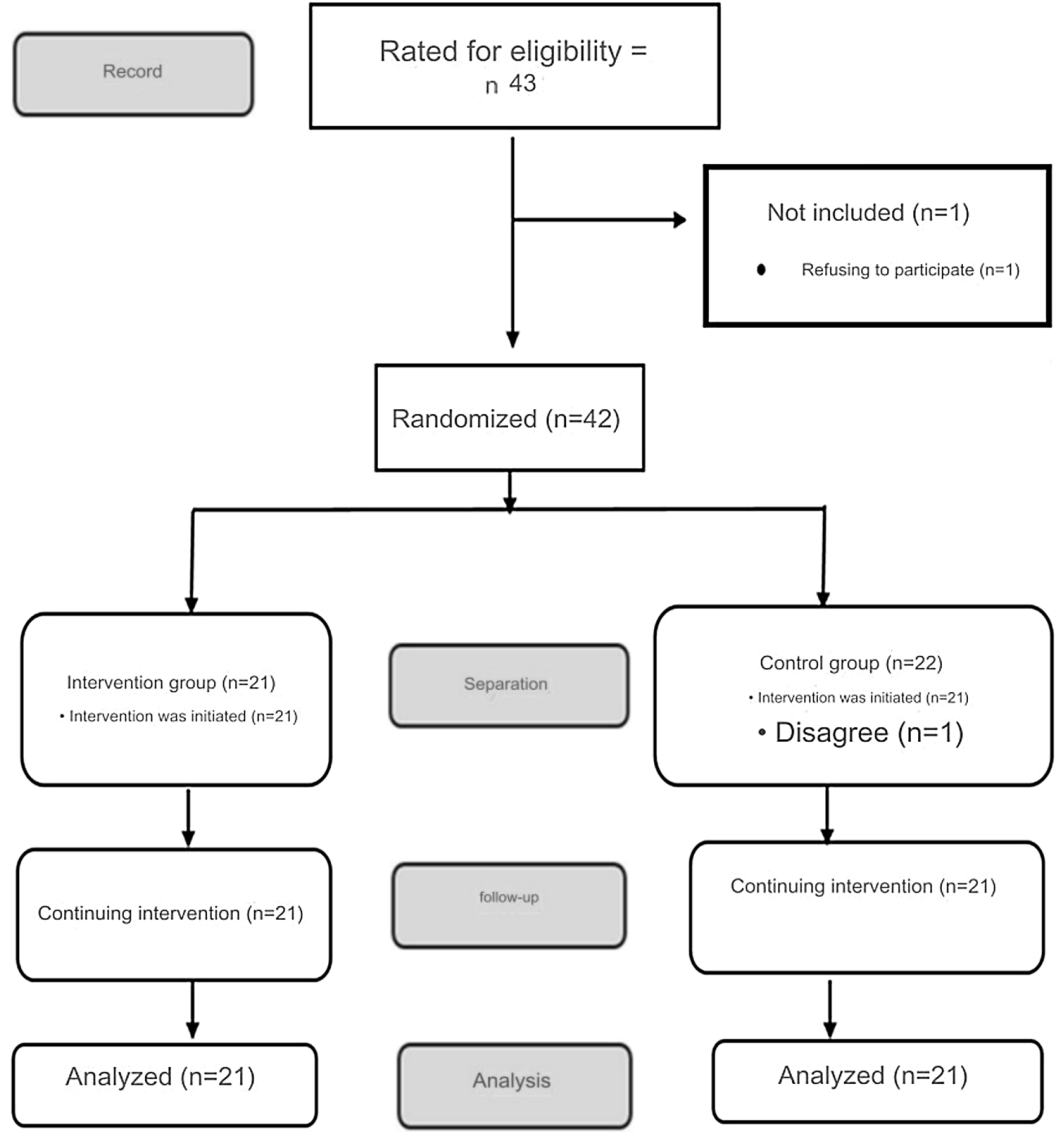
CONSORT Flowchart of Research

A total of 42 patients, 21 intervention and 21 control groups, were included in the study (Fig. 1).

When the participants analysed (Table 1), it seen that the mean ages were similar, housewives (intervention 81% and control 66.6%) and primary school graduates (intervention 47.6% and control 52.4%) were in the majority in both groups (*p* > 0.05). In addition, the groups had low socio-economic level (intervention 71.5% and control 95.2%) (*p* > 0.05). The majority of the participants had chronic diseases (intervention 76.2% and control 71.4%) (*p* > 0.05).

**Table 1:** Distribution of The Participants According to Their Groups and Demographic Characteristics

Variables		Intervention group		Control group		X^2^	p^a^
		n	%	n	%		
Marital status	Married	11	52.4	18	85.7	5.469	.054
	Single	2	9.5	0	0.0		
	Woman who lost her husband / Woman separated from her husband	8	38.1	3	14.3		
Family type	Nuclear family	14	66.7	18	85.7	2.626	.336
	Extended family	5	23.8	3	14.3		
	Other	2	9.5	0	0.0		
Place of residence	City	17	81.0	20	95.2	2.278	.409
	District	2	9.5	0	0.0		
	Village	2	9.5	1	4.8		
Education status	Illiterate	3	14.3	2	9.5	4.021	.457
	Primary school graduate	10	47.6	11	52.4		
	Secondary school graduate	0	0.0	3	14.3		
	High school graduate	6	28.6	3	14.3		
	University and above	2	9.5	2	9.5		
Employment status	Working	4	19.0	7	33.3	1.109	.292
	Not working	17	81.0	14	66.7		
Profession	Housewife	17	81.0	14	66.6	6.333	.073
	Worker	0	0.0	5	23.8		
	Officer	3	14.2	1	4.8		
	Self-employment	1	4.8	1	4.8		
Income status	My income is less than my expenses	15	71.5	20	95.2	3.978	.108
	My income is equal to my expenses	4	19.0	1	4.8		
	My income is more than my expenditure	2	9.5	0	0,0		
Chronic illness	Yes	16	76.2	15	71.4	0.123	.726
	No	5	23.8	6	28.6		
Hypertension	Yes	11	52.4	11	52.4	0.000	1.000
	No	10	47.6	10	47.6		
Diabetes Mellitus	Yes	6	28.6	4	19.0	0.525	.469
	No	15	71.4	17	81.0		
Heart disease	Yes	5	23.8	5	23.8	0.000	1.000
	No	16	76.2	16	76.2		
Immune system disease	Yes	5	23.8	4	19.0	0.141	.707
	No	16	76.2	17	81.0		
Surgery history	Evet	15	71.4	10	47.6	2.471	.116
	Hayır	6	28.6	11	52.4		
Operation name	Those without a history of surgery	6	28.5	11	52.3	8.729	.337
	Polypectomy	1	4.8	0	0.0		
	Gastro Intestinal System Surgery	2	9.5	1	4.8		
	Caesarean section	5	23.8	1	4.8		
	Myomectomy	2	9.5	0	0.0		
	Mastectomy	1	4.8	2	9.5		
	Other gynaecological surgery	1	4.8	2	9.5		
	Lumbar hernia	3	14.3	3	14.3		
	Appendicitis	0	0.0	1	4.8		
Smoking	Yes	4	19.0	8	38.1	1.867	.172
	No	17	81.0	13	61.9		
Alcohol use	Yes	3	14.3	7	33.3	2.100	.147
	No	18	85.7	14	66.7		
Total		**21**	**100.0**	**21**	**100.0**		

*X*^2^ chi-square test

^a^*p* < .05

It seen that the marital status variable showed homogeneous distribution according to the groups of the participants (*p* > 0.05). When the participants’ history of surgery was analyzed, it seen that 71.5% of the intervention group participants and 47.7% of the control group participants had a history of surgery (Table 1).

When the age distributions of the participants examined, it seen that the intervention group participants were 57.62 ± 10.53 years old on average; the control group participants were 58.19 ± 12.13 years old on average (Table 2).

**Table 2:** Age, Height and Weight Distribution of The Participants (*n* = 42)

Variables	Group	Min	Max	Average	Standard deviation	*t*	*p*^a^
Age	Intervention	41	83	57.62	10.53	-0.163	.871
	Control	35	84	58.19	12.13		
Height	Intervention	143	175	160.48	6.82	0.936	.355
	Control	150	170	158.62	6.00		
Weight	Intervention	52	127	79.62	16.94	1.202	.236
	Control	48	108	73.81	14.25		
Number of cigarette use	Intervention	0	20	1.95	5.10	-1.955	.058
	Control	0	30	6.52	9.42		
Number of living children	Intervention	0	6	2.00	1.76	-0.702	.487
	Control	0	6	2.33	1.27		

*t* independent groups t test; *Max* maximum; *Min* minimum

^a^*p* < .05

It was observed that the use of patient-controlled analgesics and narcotic analgesics was lower in women who practiced mandala (Table 3).

**Table 3:** Participants’ Use of Postoperative Analgesia

Type of post op analgesic	Parasetamol	Evet	18	85.7	0	0.0	3.231	0.232
		Hayır	3	14.3	21	100.0		
	Patient Controlled Analgesia (PCA)	Evet	1	4.8	10	47.6	9.977	0.002^a^
		Hayır	20	95.2	11	52.4		
	Narcotic Analgesia	Evet	1	4.8	7	33.3	5.559	0.045^a^
		Hayır	20	95.2	14	66.7		
Number of analgesia received from the postoperative period until post op day 2	1		3	14.3	0	0.0	3.326	0.190
	2		6	28.6	8	38.1		
	3 and more		12	57.1	13	61.9		

*X*^2^ chi-square test

^a^*p* < .05

The STAI-I preoperative scores of the control group participants were higher than those of the intervention group participants (*p* < 0.05). There was no statistically significant difference between the VAS preoperative scores of the participants according to their groups (*p* > 0.05) (Table 4).

**Table 4:** Comparison of Preoperative Scale Scores According to The Groups of Participants

	Intervention group		Control group		*T*	*p*^a^
	Mean	SD	Mean	SD		
STAI-I preoperative	56.33	7.65	63.48	5.31	-3.515	.001
VAS preoperative	0.77	1.46	0.55	1.22	0.515	.609

*t* independent groups t test; *SD* standard deviation

^a^*p* < .05

**Fig. 2:**
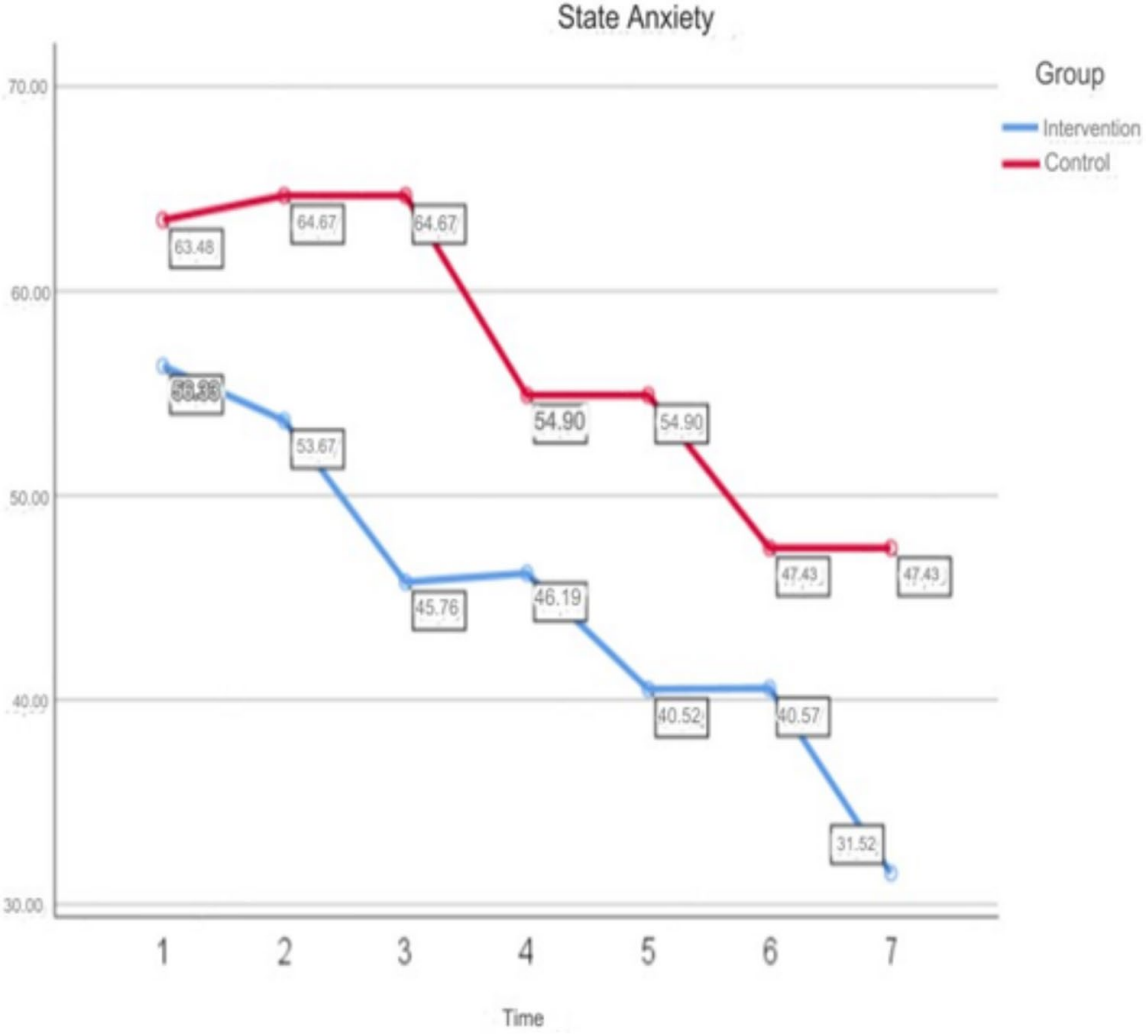
Change of STAI-I Scores of Both Groups According to Time on The Same Graph

The comparison of STAI-I scores according to group, time and group*time interaction evaluated using “Two-Way Analysis of Variance in Repeated Measures”. As a result of the analysis, a statistically significant difference found between the mean scores in terms of time (*p* < 0.05) and 71.5% of the change explained by time. There was a statistically significant difference between the mean scores in terms of group*time interaction (*p* < 0.05) and 18.7% of the change explained by group*time interaction. There is a statistically significant difference between the mean scores in terms of group (*p* < 0.05) and 59.6% of the change is explained by group (Table 5). Figure 2 shows the change in STAI-I scores of both groups over time in the same graph.

**Table 5:** In-Group Comparisons of All STAI-I Scores of Participants

Variables	Intervention group		Control group	
	Mean	SD	Mean	SD
STAI-I preoperative^a^	56.33	7.65	63.48	5.31
STAI-I postoperative 8th hour before mandala activity^b^	53.67	6.24	64.67	3.94
STAI-I postoperative 8th hour after mandala activity^c^	45.76	5.92	64.67	3.94
STAI-I postoperative day 1 post op before mandala activity^d^	46.19	7.26	54.90	6.89
STAI-I postoperative day 1 post op after mandala activity^e^	40.52	7.92	54.90	6.89
STAI-I postoperative day 2 post op before mandala activity^f^	40.57	7.95	47.43	8.29
STAI-I postoperative day 2 post op after mandala activity^g^	31.52	6.73	47.43	8.29
*F*	39.681		102.322	
*p*	.001		.001	
Bonferroni	a,b > c,d,e,f,g c > f,g d,e,f > g		a,b,c > d,e,f,g	
*F* (time)	*F* = 108.487 p = .001 Effect size = 0.715			
*F* (group*time)	*F* = 9.181 p = .001 Effect size = 0.187			
*F* (group)	*F* = 58.915 p = .001 Effect size = 0.596			

*F* ANOVA test; *SD* standard deviation

*p* < .05

There was a statistically significant difference between the VAS all measurement scores of the intervention group participants (*p* < 0.05). As a result of the Bonferroni test to determine from which measurement the difference originated; it seen that the VAS preoperative scores of the intervention group participants were lower than the other measurements (Table 6).

**Table 6:** In-Group Comparisons of All VAS Scores of Participants

Variables	Intervention group		Control group	
	Mean	SD	Mean	SD
VAS preoperative^a^	0.77	1.46	0.55	1.22
VAS postoperative 8th hour before mandala activity (time: 22:00)^b^	6.68	2.11	7.17	1.42
VAS postoperative 8th hour after mandala activity (time: 2300)^c^	5.78	2.26	7.17	1.42
VAS postoperative 8th hour after mandala activity (time: 02:00)^d^	5.12	1.98	6.45	1.50
VAS postoperative 8th hour after mandala activity (time: 06:00)^e^	4.71	2.09	6.10	1.53
VAS postoperative day 1 before mandala activity (time: 10:00)^f^	5.13	2.15	5.59	1.62
VAS postoperative day 1 after mandala activity (time: 11:00)^g^	4.03	2.28	5.59	1.62
VAS postoperative day 1 after mandala activity (time: 14:00)^h^	4.24	2.12	4.96	1.59
VAS postoperative day 1 after mandala activity (time: 18:00)^i^	3.98	2.12	4.25	1.74
VAS postoperative day 1 after mandala activity (time: 22:00)^j^	3.81	2.09	3.69	1.72
VAS postoperative day 2 after mandala activity (time: 02:00)^k^	4.04	2.29	2.75	2.12
VAS postoperative day 2 after mandala activity (time: 06:00)^l^	3.19	1.92	2.18	2.21
VAS 2nd postoperative day before mandala activity (time: 10:00)^m^	3.10	2.04	1.77	1.88
VAS 2nd postoperative day after mandala activity (time: 11:00)^n^	2.24	1.89	1.77	1.88
VAS 2nd postoperative day after mandala activity (time: 14:00)^o^	1.77	1.79	0.82	1.46
*F*	33.128		118.274	
*p*	.001		.001	
Bonferroni	a < b,c,d,e,f,g,h,ı,i,k,l,m b > d,e,f,g,h,ı,i,k,l,m,n,o c > g,h,ı,i,k,l,m,n,o d > ı,i,k,l,m,n,o e,f > l,m,n,o g,h,ı,i,k,l,m > n,o		a < b,c,d,e,f,g,h,ı,i,k, b,c > e,f,h,ı,i,k,l,m,o d,e > h,ı,i,k,l,m,o f,g > h,ı,i,k,l,m,o h > ı,i,k,l,m,o ı,i > k,l,m,o	
*F* (time)	F = 122.333 p = .001 Effect size = 0.754			
*F* (group*time)	F = 8.826 p = .001 Effect size = 0.181			
*F* (group)	F = .097 p = .757			

*F* ANOVA test, *SD* standard deviation

*p* < .05

The comparison of VAS scores according to group, time and group*time interaction was evaluated using “Two-Way Analysis of Variance in Repeated Measures” (Fig. 2). As a result of the analysis, there was a statistically significant difference between the mean scores in terms of time (*p* < 0.05) and 75.4% of the change was explained by time. There is a statistically significant difference between the mean scores in terms of group*time interaction (*p* < 0.05) and 18.1% of the change is explained by group*time interaction. There is no statistically significant difference between the mean scores in terms of group (*p* > 0.05) (Table 6).

**Fig. 3:**
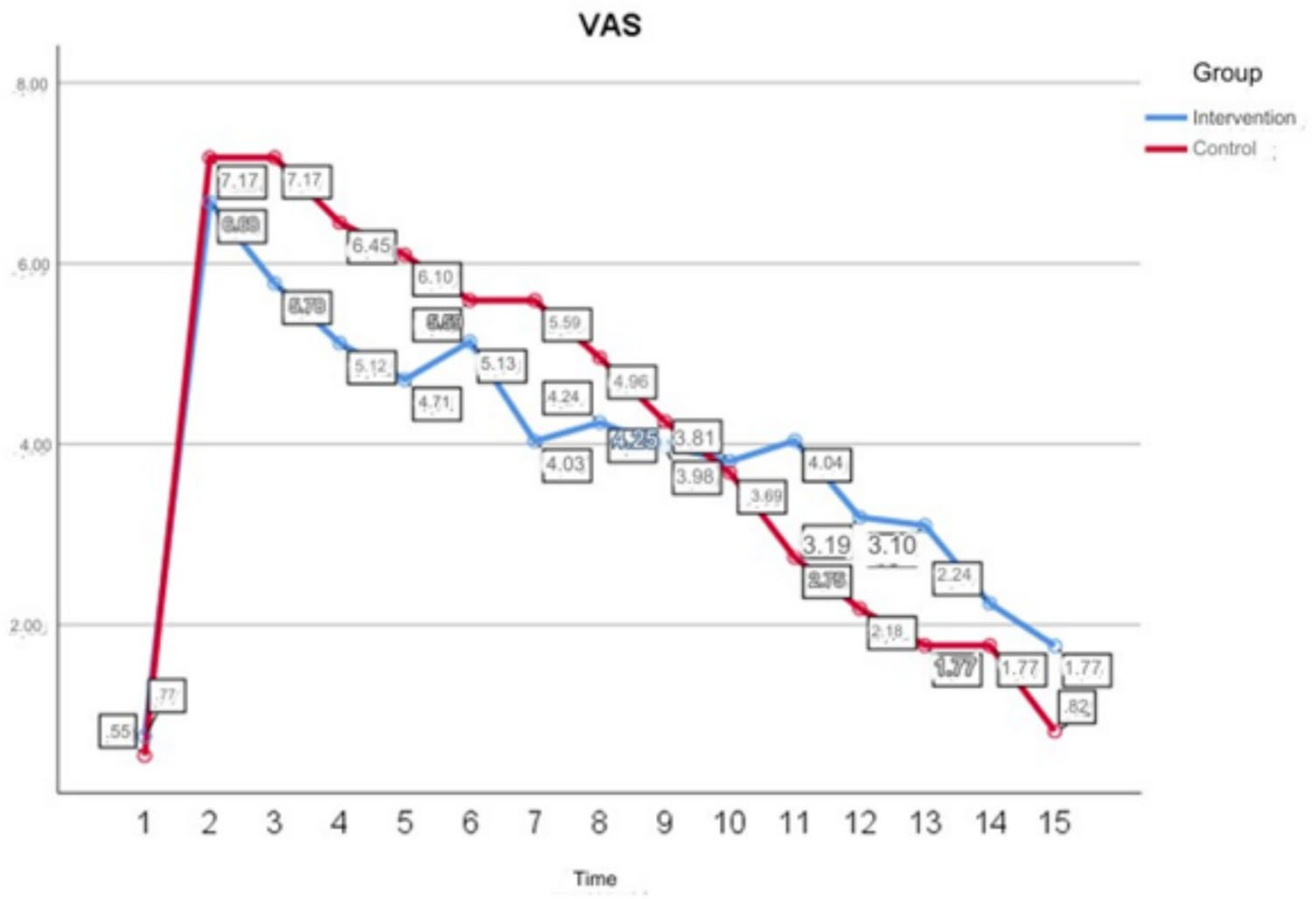
Change Graph of VAS Scores of Both Groups According to Time

It is seen that 81% of the participants in the intervention group enjoyed the mandala activity and thought that the mandala activity relaxed them a little bit (Fig. 3).

## Discussion

This study conducted to investigate the effect of mandala activity on postoperative pain and anxiety levels in gynecologic oncology patients. It observed that women who practiced mandala activity at least once a day after surgery had reduced pain and anxiety levels. Additionally, it observed that the analgesia application also affected the type of analgesia.

In a study in which preoperative anxiety of surgical patients measured with STAI-I, the mean STAI-I scale score was 36.8 ± 12.63. The same study showed that anxiety may differ according to educational level, age and the reason of the operation [28]. In this study, the STAI scale score was calculated as 56.33 ± 7.65 in the intervention group and 63.48 ± 5.31 in the control group before surgery.

In a thesis study on fear of pain conducted in a general surgery service, it observed that the VAS score before surgery was 3.36 ± 3.48 [29]. Preoperative VAS scores were 0.77 ± 1.46 in the intervention group and 0.55 ± 1.22 in the control group. Preoperative state anxiety levels were higher in the control group than in the intervention group, while no difference was observed in pain status. No study found in the literature that measured pain and anxiety together preoperatively in gynaecological oncology surgery. It observed that similar type of studies were in minority in the literature and there was a correlation between pain and anxiety in surgery.

It is stated in the literature that mandala activity has an anxiety-reducing effect [30]. In another pilot study conducted with mandala activity, the STAI score before mandala was 44.38 ± 16.96 and the STAI score after mandala activity was 29.46 ± 9.10. In a study conducted with women in the third trimester, It observed that mandala activity reduced anxiety [31]. In Gürcan’s study with adolescents undergoing cancer treatment, it determined that mandala activity significantly reduced anxiety [32]. In the study in which art therapy applied with cancer patients undergoing chemotherapy (44% breast cancer, 22% gastrointestinal cancer, 18% haematological cancer and 20% other cancer), it found that all VAS measurements decreased and art therapy reduced anxiety [33]. In the study of Yakar et al., it observed that anxiety levels of 12 breast cancer patients decreased after mandala activity [9]. In another study, mandala therapy was administered to breast cancer patients, and their STAI scores were measured before and after chemotherapy. It was observed that the scores of participants in the intervention group decreased, while those in the control group remained unchanged [34]. In a meta-analysis study on mandala in the literature, it determined that the majority of the studies used the STAI scale and most of the studies were single-session. In addition, in the meta-analysis study, it determined that mandala activity significantly reduced anxiety [12]. In a study of infertile women undergoing embryo transfer, mandala activity with meditation and music therapy was found to reduce anxiety [35]. Studies in the literature conclude that mandala has a positive effect on anxiety.

Our findings regarding state and trait anxiety levels in patients undergoing postoperative care after gynecologic oncology surgery can be placed in the context of existing research on peri-operative anxiety. For example, in a study of laparoscopic gynecologic surgery patients (*n* = 330), higher preoperative anxiety (measured on the APAIS rather than STAI) was associated with higher postoperative pain scores and increased analgesic requirement [36]. Another review focusing on gynecological oncology found that peri-operative and postoperative anxiety constitute a significant, yet under-addressed, component of care in this patient population [37]. Furthermore, a study on women with gynecologic cancers found that mandala art therapy reduced anxiety levels post-intervention [20]. In a study in which reflexology was studied with hysterectomy patients, the preoperative STAI score of the intervention group was 40.17 ± 2.72 and the postoperative STAI score was 31.00 ± 2.69. In a study conducted with women undergoing hysterectomy surgery, the mean STAI-I score applied after surgery was found to be 44.10 ± 10.62 [38]. The findings from Gu et al. (2023) show that patients with elevated preoperative anxiety had significantly worse pain (VAS) and required higher rescue analgesia in the 48 h post-surgery [36]. In this study, the anxiety level of the control group before surgery was higher than the intervention group before and after the mandala activity at the 8th hour after surgery. In the intervention group, the state anxiety score was higher before the mandala activity at the 8th hour postoperatively than after the mandala activity. When the postoperative first day state anxiety scores were analysed, it seen that the anxiety level was higher in the control group before the mandala activity. In the intervention group, higher anxiety level was observed before the mandala activity than after the mandala activity. On the second postoperative day, the anxiety level of the control group was higher than the intervention group before the mandala activity. In the intervention group, the anxiety level was higher before the mandala activity. In this study, It observed that the state anxiety of the intervention group decreased after the mandala activity. In the control group, state anxiety was found to be higher than the intervention group. It seen that mandala activity decreased state anxiety over time. According to the literature, it is seen that mandala activity reduces the level of state anxiety in different types of researches. With this research, the hypothesis ‘The application of mandala activity in the postoperative period of gynaecological oncology patients reduces their state anxiety levels’ was confirmed.

The VAS score after a thesis operation performed in the general surgery department regarding fear of pain was found to be 5.17 ± 2.72 (preoperative VAS score: 3.36 ± 3.48) [29]. In a study where reflexology, a non-pharmacological method, was studied with patients who had hysterectomy, it seen that the pain score of the control group was 1.02 ± 2.55 before surgery and the pain score of the intervention group was 0.85 ± 2.11 before surgery. After the reflexology sessions and at the 48th hour after the surgery, the control group pain score was 3.35 ± 1.73 and the pain score for the noted categories was 0.94 ± 1.50. In the study conducted with women who underwent hysterectomy surgery, it seen that the VAS score applied after the surgery was 5.52 ± 1.20 [38] In this study, VAS scores were compared at intervals before and after the operation, and time and group modeling revealed that the intervention group performing mandibular activity showed a significant difference in VAS scores (*p* = 0.001).

The limitation of this study is that the mandala activity was implemented in booklet form and the mandalas in the booklet were arranged in the same order. This situation led to the mandalas on the first pages of the booklet being colored more frequently by patients during the mandala activity. Another limitation of the study is that it was conducted in a single hospital. The strengths of this study are that there are limited studies in the literature on mandala activity and pain [21].

Midwives, as key providers of women-centered care, can utilize these findings to strengthen their role in gynecological cancer prevention and awareness. Incorporating counseling on early warning signs, risk factors, and screening behaviors into routine midwifery practice may enhance women’s health outcomes. Moreover, integrating gynecological oncology awareness into midwifery education and clinical guidelines could further expand the preventive and supportive scope of midwifery services.

## Conclusion

As a result of the analysis obtained from the data of this study, it concluded that mandala activity decreased the postoperative pain and anxiety levels of gynecologic oncology patients and affected the use of analgesia. It observed that women with gynecological cancer most frequently applied to the hospital due to bleeding. While patient-controlled analgesia (PCA) and narcotic analgesia were observed to be used more frequently in the control group than in the intervention group, paracetamol and Non-Steroidal Anti-Inflammatory Drugs-derived analgesia were observed to be used more frequently in the intervention group. It is also important for women’s health that midwives have more knowledge about mandala activity and integrate it into gynecological oncology as a non-pharmacological and adjuvant treatment. In addition, it is recommended that studies involving more and diverse sample groups be conducted in order to evaluate the research findings in the literature. This study underscores the relevance of gynecological oncology within midwifery practice. Enhancing midwives’ knowledge and competencies in this area can contribute to early detection and improved outcomes in women’s health, reinforcing the preventive and holistic nature of midwifery care.

## Data Availability

The data that support the findings of this study are available from the authors.
